# Simultaneous blockade of AP-1 and phosphatidylinositol 3-kinase pathway in non-small cell lung cancer cells

**DOI:** 10.1038/sj.bjc.6604782

**Published:** 2008-11-18

**Authors:** J Kikuchi, I Kinoshita, Y Shimizu, S Oizumi, M Nishimura, M J Birrer, H Dosaka-Akita

**Affiliations:** 1 First Department of Medicine, Hokkaido University School of Medicine, Sapporo, Japan; 2 Department of Medical Oncology, Hokkaido University Graduate School of Medicine, Sapporo, Japan; 3 Cell and Cancer Biology Branch, National Cancer Institute, Bethesda, MD 20892, USA

**Keywords:** AP-1, phosphatidylinositol 3-kinase pathway, non-small cell lung cancer, LY294002, TAM67

## Abstract

c-Jun is a major constituent of AP-1 transcription factor that transduces multiple mitogen growth signals, and it is frequently overexpressed in non-small cell lung cancers (NSCLCs). Earlier, we showed that blocking AP-1 by the overexpression of a c-Jun dominant-negative mutant, TAM67, inhibited NSCLC cell growth. The phosphatidylinositol 3-kinase (PI3K)/Akt signal transduction pathway is important in transformation, proliferation, survival and metastasis of NSCLC cells. In this study, we used NCI-H1299 Tet-on clone cells that express TAM67 under the control of inducible promoter to determine the effects of inhibition of AP-1 and PI3K on cell growth. The PI3K inhibitor, LY294002, produced a dose-dependent inhibition of growth in H1299 cells and that inhibition was enhanced by TAM67. TAM67 increased dephosphorylation of Akt induced by LY294002 and reduced the TPA response element DNA-binding of phosphorylated c-Jun. TAM67 increased G1 cell cycle blockade induced by LY294002, which was partially associated with cyclin A decrease and p27^Kip1^ accumulation. Furthermore, TAM67 and LY294002 act, at least additively, to inhibit anchorage-independent growth of the H1299 cells. These results suggest that AP-1 and PI3K/Akt pathways play an essential role in the growth of some NSCLC cells.

*c-jun* is the cellular homologue of the oncogene *v-jun* that was originally identified as the transforming sequence of avian sarcoma virus 17. c-Jun is also a central component of AP-1 that consists of homodimers and heterodimers of the Jun, Fos and ATF gene family members, and it regulates transcription through AP-1 and cAMP responsive elements (Angel *et al*, 1987, 1988; Curran and Franza, 1988; Sassone-Corsi *et al*, 1990). Although the role of c-Jun in human cancers remains to be defined, substantial evidence suggests that it is involved in cellular proliferation and transformation. Deregulated expression of c-Jun induces immortalised rat fibroblasts to grow in an anchorage-independent fashion (Schütte *et al*, 1989) depending on the induction of multiple c-Jun target genes (Kinoshita *et al*, 2003; Leaner *et al*, 2003, 2005; Hommura *et al*, 2004; Katabami *et al*, 2005). Recent studies reported that specific AP-1 blockade by a dominant-negative mutant of c-Jun, TAM67, inhibited the growth of some types of human cancer cells by causing G1 arrest (Ludes-Meyers *et al*, 2001; Liu *et al*, 2004; Suto *et al*, 2004).

Earlier studies (Wodrich and Volm, 1993; Szabo *et al*, 1996) suggested that c-Jun had a role in early events in the pathogenesis of lung cancers because it was highly expressed in 31–50% of patients with non-small cell lung cancers (NSCLCs), and it was also upregulated in atypical bronchial epithelium. In a previous study, we showed that TAM67 inhibited lung cancer growth both *in vivo* and *in vitro* using NCI-H1299 (H1299) NSCLC cells that expressed TAM67 under the control of an inducible promoter that blocked AP-1 activity (Shimizu *et al*, 2008). Taken together with its transforming properties, c-Jun may have pivotal roles in lung carcinogenesis and lung cancer growth.

The PI3K/Akt signal transduction pathway regulates various cellular processes including transformation, proliferation, survival and metastasis in a variety of cancer cells, including lung cancer cells (Kennedy *et al*, 1999; Brodt *et al*, 2000; Vivanco and Sawyers, 2002). Clear evidence shows that the PI3K/Akt signalling pathway is involved in lung carcinogenesis (Brognard *et al*, 2001; Chun *et al*, 2003; Lee *et al*, 2003; Tsao *et al*, 2003; Massion *et al*, 2004). Akt is frequently activated in both premalignant human bronchial epithelial cells and NSCLC cells. Akt activation may be an early event in lung tumorigenesis (West *et al*, 2003). Recent studies reported that the inhibition of PI3K activity by LY294002 reduced human cancer cell growth *in vivo* and *in vitro* by apoptosis or G1 cell cycle arrest (Casagrande *et al*, 1998; Hu *et al*, 2000; Semba *et al*, 2002; Gao *et al*, 2004; Takeda *et al*, 2004; Sourbier *et al*, 2006).

In this study, we investigated the antiproliferative effects of the inhibition of AP-1 and PI3K, alone and together, in the H1299 Tet-on clone cells using c-Jun dominant-negative mutants, TAM67 and LY294002.

## Material and methods

### Cell lines, culture conditions

The human NSCLC cell lines, H1299, that expressed either TAM67 (H1299-TAM67) or green fluorescent protein (H1299-GFP) in a doxycycline-controlled manner were described previously (Shimizu *et al*, 2008). Briefly, pLRT-TAM67 and -GFP were transfected into packaging Phoenix A cells by calcium phosphate transfection, and retroviruses were harvested after 48 h of transfection and infected into H1299 cells (Watsuji *et al*, 1997). Stable transfectants were selected using 5 *μ*g ml^−1^ blasticidin (Invitrogen, Life Technologies Inc., Carlsbad, CA, USA) and screened by western blot analysis for inducibility of TAM67 expression in response to 2 *μ*g ml^−1^ doxycycline. We chose clones TAM67 nos.8, 34 and 38 because of their highly inducible TAM67 expression in the presence of doxycycline. As controls, we used clones GFPs, 5 and 8 that had no inducible TAM67 expression.

Cells were cultured in RPMI 1640 (Invitrogen Life Technologies Inc.) medium supplemented with 10% foetal bovine serum (FBS) and 0.03% glutamine at 37°C in an atmosphere of 5% CO_2_. LY294002 (Sigma-Aldrich Co., St Louis, MO, USA) was dissolved in DMSO at 10 mM and used at final concentration of 0.5–20 *μ*M.

### Cell growth assays

Cells were seeded at 2500–5000 cells per well in 96-well plates in normal growth medium with or without 2 *μ*g ml^−1^ doxycyclin for 24 h, followed by the addition of 0, 2.5, 5 or 20 *μ*M of LY294002 for 3 days. Anchorage-dependent growth was measured in 96-well plates using an MTT (dimethyl thiozolyl-2′,5′-diphenyl-2-*H*-tetrazolium bromide)-based assay (non-radioactive proliferation assay, Promega Corp., Madison, WI, USA) as described previously (Sabichi *et al*, 1998).

Anchorage-independent growth assays were performed using 0.4% soft agarose (Seaplaque, FMC Corp., Rockland, ME, USA) in 6-well plates with or without 0.1 *μ*g ml^−1^ doxycyclin and LY294002 (0.5, 1 or 2.5 *μ*M) as described previously (Sabichi *et al*, 1998). After 2 weeks of incubation, colonies were stained with p-iodonitrotetrazolium violet (Sigma-Aldrich Co.) and counted using NIH Image ver 1.62 software (NIH, Bethesda, MD, USA).

### Western blotting

Cell lysates from H1299 Tet-on clone cells grown in the absence or presence of doxycycline (2 *μ*g ml^−1^) in combination with LY294002 were prepared by lysing the cells in radioimmune precipitation assay buffer (150 mM NaCl, 1% Triton X-100, 1% deoxycholate, 0.1% SDS, 10 mM Tris (pH 7.4)) supplemented with 100 *μ*g ml^−1^ leupeptin, 100 *μ*g ml^−1^ aprotinin and 10 mM phenylmethylsulfonyl fluoride. The cell lysates were sonicated and centrifuged to remove debris, and protein concentrations were determined using the Bio-Rad Protein Assay kit (BioRad Laboratories, Hercules, CA, USA). Equal amounts of protein were separated on 12 or 15% SDS gels, transferred with nitrocellulose membranes (Amersham Biosciences Inc. St Albans, UK), and incubated with the following antibodies: anti-p27^kip1^ (610242; BD Transduction Laboratories, KY, USA), anti-cyclin D1 (sc-246, Santa Cruz Biotechnology, Santa Cruz, CA, USA), anti-cyclin E (sc-247, Santa Cruz Biotechnology), anti-cyclin A (sc-751, Santa Cruz Biotechnology), anti-phosphorylated-Akt (Ser473; no. 9271, Cell Signaling Technology, Beverly, MA, USA), anti-Akt, (no. 9272, Cell Signaling Technology). Total cell extracts from Jurkat cells prepared with or without LY294002 were used as a positive or negative control of the assay for phosphorylated-Akt. The primary antibodies were detected using antirabbit or antimouse antibody conjugated with horseradish peroxidase (NA934V, NA931V, Amersham Biosciences Inc., St Albans, UK), and visualised using the Amersham ECL system after washing with TBST six times (5 min each) after the incubation of the first and second antibodies. The intensity of the bands after western blotting was determined by laser scanning of the films followed by quantitative densitometric analysis using NIH Image Ver 1.62 software. Standardisation was with actin measured in the same blots with antiactin antibody (A-2066, Sigma-Aldrich Co.).

### Cell cycle analysis

H1299 Tet-on clone cells were cultured in 100-mm plates with or without 2 *μ*g ml^−1^ doxycyclin for 24 h, followed by the addition of 0, 5 or 20 *μ*M LY294002 for 3 days. Then, cells were trypsinized, washed twice with PBS and fixed in 70% ethanol at −20°C. Fixed cells were centrifuged and resuspended in 250 *μ*g ml^−1^ RNase and 50 *μ*g ml^−1^ propidium iodide (Sigma) for DNA staining. DNA content was measured by a FACScan flow cytometer (Becton Dickinson, San Jose, CA, USA) and two software packages: CellQuest 3.1 (BD Pharmingen, San Diego, CA, USA) and ModFit LT 2.0 (Verity Software House, Topsham, ME, USA).

### c-Jun activation assay

Nuclear protein extracts were obtained from cell cultures using the nuclear extract kit (Active Motif, CA, USA) according to the manufacturer's instruction. The activation of c-Jun was measured using the TransAM™ AP-1 family transcription assay kit (Active Motif) according to the manufacturer’s instruction (Debinski and Gibo, 2005; Polytarchou *et al*, 2005; Shimizu *et al*, 2008). This method measures the DNA-binding activity of AP-1 by ELISA. Briefly, 2.5 *μ*g of nuclear protein samples were incubated for 1 h in a 96-well plate coated with an oligonucleotide containing a TPA response element (TRE; 5′-TGAGTCA-3′) that specifically binds with phosphorylated c-Jun (p-c-Jun) contained in nuclear extracts. For specificity control, an excess amount (20 pmol) of mutant probe was added to the reaction in a competition assay. After washing, p-c-Jun antibody (1 : 500 dilutions) was added to these wells and incubated for 1 h. Following incubation for 1 h with a secondary HRP-conjugated antibody (1 : 1000 dilution), specific binding was detected by colorimetric estimation at 450 nM with a reference wavelength of 655 nM. Note that the antibody against c-Jun recognises phosphorylated serine 73 of the transactivating domain of c-Jun and does not detect TAM67, in which most of the transactivating domain is deleted. Nuclear extracts from K562 cells stimulated by TPA were used as a positive control of the assay for c-Jun.

### Statistical analysis

All values are presented as mean±s.d. Statistical significance was determined using Student's unpaired, two-tailed *t*-test.

## Results

### Induction of TAM67 enhanced antiproliferative effect of PI3K inhibitor LY294002

**Figure 1:**
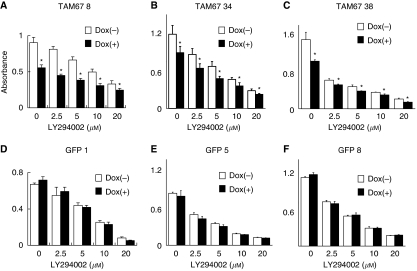
Effects on the cell growth of LY294002, TAM67 and their combination. (**A**–**C**) H1299-Tet-on-TAM67 clone cells (TAM67 nos. 8, 34, and 38). (**D**–**F**) H1299-Tet-on-GFP clone cells (GFPs 1, 5 and 8). The H1299 clones were treated with solvent alone (0), or the indicated doses of LY294002 with or without TAM67 induction for 72 h at which time they were subjected to 3-(4,5-dimethylthiazol-2-yl)-2,5-diphenyltetrazolium bromide assays. Data points are the mean±s.d. of quadruplet samples in representative one of the three independent experiments. ^*^P<0.01 compared with cells treated with each concentration of LY294002 alone.

Earlier, we showed that the induction of TAM67 inhibited anchorage-dependent growth in H1299 clones that express TAM67 (Shimizu *et al*, 2008). In this study, we investigated the growth inhibition of H1299 cells treated with LY294002 in combination with the induction of TAM67 (Figure 1A–F). LY294002 produced a dose-dependent inhibition of the growth of H1299 cells in the MTT assay. The induction of TAM67, but not GFP, enhanced the growth inhibition of LY294002 in the H1299 Tet-on clone cells. These results suggest that LY294002 and TAM67 have at least an additive effect on cell growth inhibition.

### The induction of TAM67 enhances dephosphorylation of Akt by LY294002

**Figure 2:**
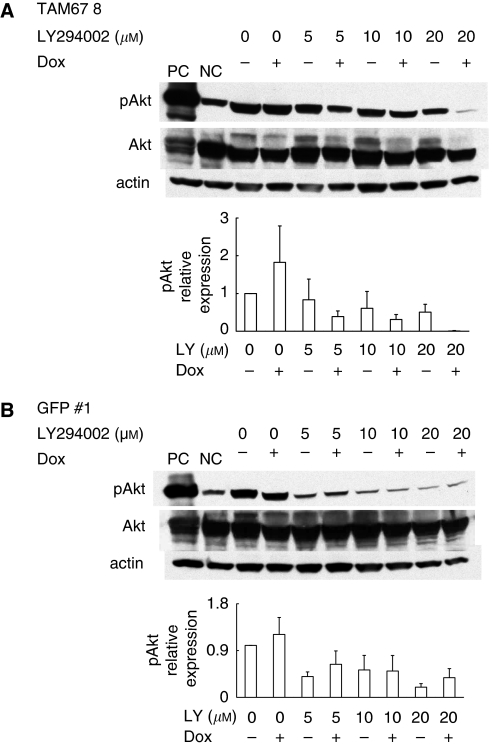
Effects on PI3K/Akt pathway activation of LY294002, TAM67 and their combination in H1299 cells. (**A**) H1299-Tet-on-TAM67 clone cells (TAM67 no. 8) and (**B**) H1299-Tet-on-GFP clone cells (GFP 1). Western blots of cell lysates incubated with antibodies against phospho-Akt (S473) and Akt in cells treated with LY294002, the induction of TAM67 and their combination. Representative radiographs of three independent experiments. Standardisation was with actin measured in the same blots. Comparison of protein expression levels among the various conditions is based on the ratio of expression of a protein in each condition to that in non-treated one (set equal to 1). NIH image 1.62 software was used to densitise and quantify the amount of the bands. The data show the average value of three independent experiments with error bars representing s.d. PC, positive control; NC, negative control.

LY294002 inhibited the phosphorylation of Akt (Ser473) in a dose-dependent manner (Figure 2). Interestingly, TAM67 further decreased the phosphorylation of Akt at 5–20 *μ*M of LY294002, whereas TAM67 alone did not show such effects. This observation suggests that TAM67 and LY294002 may have a synergistic effect on inhibiting Akt activity.

### TAM67 inhibited AP-1 activity

**Figure 3:**
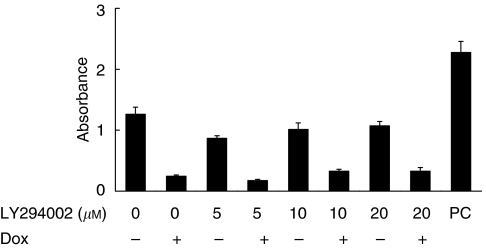
DNA-binding activity of p-c-Jun in TAM 8 analysed by the TransAM AP-1 family transcription assay kit. TAM67 reduced the binding of phosphorylated c-Jun to TRE. Each value represents the mean±s.d. of triplicated samples in representative one of three independent experiments. PC, positive control.

We determined the effects of single or combined treatment with LY294002 and TAM67 on AP-1 activity using ELISA-based TransAM™ AP-1 family transcription assay kit (Figure 3). TAM67 reduced the binding of p-c-Jun to TRE at each concentration of LY294002, whereas LY294002 did not affect the binding. These results confirmed that TAM67 inhibited AP-1 activity over a wide range of concentrations of LY294002.

### The induction of TAM67 enhances G1 cell cycle block by LY294002

**Figure 4:**
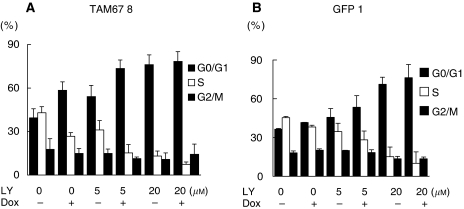
Effect on the proliferative arrest of LY294002, TAM67 and their combination. Cells were incubated with the indicated doses of LY294002 with or without induction of TAM67 for 72 h. The percentage of cells in each phase was measured by an FACS flow cytometer and analysed using ModFitLT software. (**A**) Cell cycle analysis of TAM67 no. 8. The data show the average percentage of five independent experiments with error bars representing the s.d. (**B**) Cell cycle analysis of GFP 1. The data show the average percentage of three independent experiments with error bars representing the s.d.

We used flow cytometry to determine whether enhanced growth inhibition was because of cell cycle arrest or apoptosis of H1299 clones (Figure 4A and B). Earlier, we showed that the induction of TAM67, but not GFP, induced G1 cell cycle blockade in H1299 Tet-on clone cells (Shimizu *et al*, 2008). LY294002 increased the percentage of cells in G0/G1 phase with an associated decrease in S phase. The induction of TAM67, but not GFP, enhanced G1 cell cycle block by LY294002. Sub-G1 apoptotic fraction was not observed by LY294002, TAM67 or by both (data not shown). Low-dose (5 *μ*M) LY294002 with induction of TAM67 induced G1 cell cycle block similar to that induced by high-dose (20 *μ*M) LY294002. The additive effect between LY294002 and TAM67 was more apparent at a low dose (5 *μ*M) than a high dose (20 *μ*M) of LY294002. These results suggest that LY294002 and TAM67 produced an additive inhibition in cell growth by G1 cell cycle blockade in H1299.

### High-dose LY294002 upregulates p27 expression and TAM67 decreases cyclin A expression

**Figure 5:**
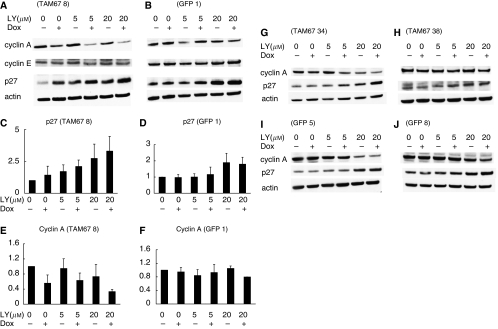
Representative western blots for cyclin A, cyclin E, p27 and actin. (**A**) TAM67 no. 8, (**B**) GFP 1, (**G**) TAM67 nos.34 and (**H**) 38, (**I**) GFP 5 and (**J**) GFP 8. (**C**–**F**) In TAM67 no.8 and GFP 1, protein expression levels among the various conditions were compared on the basis of the ratio of expression of a protein in each condition to that in non-treated one (set equal to 1). The data show the average value of six independent experiments with error bars representing the s.d. NIH image 1.62 software was used to densitise and quantify the amount of the bands.

We measured the expression of p27, cyclins A, D1 and E that control the G1–S-phase transition to investigate how TAM67 enhanced G0/G1 arrest induced by LY294002 (Figure 5). Western blot analysis showed that high-dose LY294002 treatment increased p27 in both TAM67 no.8 and GFP 1 clones (Figure 5A–D). Expression of cyclin A decreased after the induction of TAM67 in TAM67 no. 8 clone (Figure 5A and B). The increase of p27 by high-dose LY294002 and the decrease of cyclin A by TAM67 were also observed in other clones (Figure 5G–J). Although the induction of TAM67 slightly increased p27 in TAM67 no. 8 clones, this phenomenon was not observed in other TAM67 clones (Figure 5A–D and G–J). No significant changes were observed in cyclin E expression (Figure 5A and B), and cyclin D1 expression was not detected (data not shown). These results suggest that reduced expression of cyclin A by TAM67, and increased expression of p27 by high-dose LY294002 are involved in increased G1 arrest in H1299 cells.

### LY294002 and induction of TAM67 inhibit anchorage-independent growth

**Figure 6:**
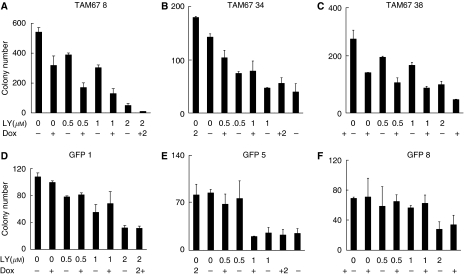
Inhibition of anchorage-independent growth by LY294002, TAM67 and their combination. Representative graphs of three independent experiments. (**A**–**C**) H1299-Tet-on-TAM67 clone cells (TAM67 nos. 8, 34 and 38). (**D**–**F**) H1299-Tet-on-GFP clone cells (GFPs 1, 5 and 8). Data are means±s.d. of triplicate samples. Similar results were obtained in three independent experiments.

We determined the effects of LY294002 and TAM67 on anchorage-independent growth (Figure 6A–F). LY294002 reduced anchorage-independent growth in a dose-dependent fashion. Induction of TAM67, but not GFP, enhanced the inhibition of anchorage-independent growth by LY294002. These results indicate that LY294002 and induction of TAM67 act, at least additively, to inhibit anchorage-independent growth of the H1299 cells.

## Discussion

This study showed the enhanced suppressive effects of a c-Jun dominant-negative mutant, TAM67 and LY294002 on both anchorage-dependent and -independent growth of a NSCLC cell line. These effects were associated with G1 cell cycle arrest, suggesting that some NSCLC cells depend on both AP-1 and PI3K/Akt pathways for cell growth.

The observed G1 cell cycle arrest was partially associated with decreased expression of cyclin A by TAM67 and increased expression of p27 by high-dose LY294002. The accumulation of p27 because of the inhibition of PI3K activity by LY294002, and its association with cell cycle arrest in the G1 phase have been shown in ovarian cancer, pancreatic ductal carcinoma and choroidal melanoma cell lines (Casagrande *et al*, 1998; Hu *et al*, 2000; Gao *et al*, 2004; Takeda *et al*, 2004). Cylin A functions during both G1-S and G2-M phases of the cell cycle (Girard *et al*, 1991; Pagano *et al*, 1992; Resnitzky *et al*, 1995). To our knowledge, the decreased expression of cyclin A by TAM67 was not reported previously, whereas TAM67 has been shown to inhibit breast cancer cell growth by reducing the expression of G1 cyclins D1 and E (Ludes-Meyers *et al*, 2001). These differences may be because of the different cell types. In immortalised rat fibroblasts, cyclin A is a direct c-Jun target gene and is necessary for c-Jun-induced anchorage-independent growth (Katabami *et al*, 2005). The increase of p27 by LY294002 and reduction of cyclin A expression by TAM67 may be involved in the enhanced antiproliferative effect of TAM67 and LY294002 when used in combination.

Using the H1299 NSCLC cells, Lee *et al* (2003, 2005) reported that PI3K/Akt and MKK4/JNK pathways cooperated to promote cell proliferation by maintaining cell survival *in vivo* and *in vitro*, and simultaneous blockade of both pathways induced apoptosis . In this study, using the same cells, blocking these pathways with LY294002 and TAM67 enhanced cell proliferative arrest more than either agent alone, but neither agent alone nor their combination induced apoptosis. Lee *et al* (2003, 2005) used JNK inhibitor, SP600215 or a dominant-negative mutant of MKK4 to inhibit MKK4/JNK pathways, whereas we used the dominant-negative mutant of c-jun, TAM67. They speculated that the MKK4/JNK inhibitor induced apoptosis because JNK directly phosphorylates Bcl-2 *in vitro* and collaborates with Bcl-2 to mediate prolonged cell survival following various stress applications (Deng *et al*, 2001). TAM67 does not have direct effect on the phosphorylation of Bcl-2, although TAM67 inhibits AP-1 activity by quenching Jun, Fos and ATF family members to inhibit not only MKK4/JNK pathway but also MEK/ERK pathway. We speculate that these differences between JNK inhibitor and TAM67 may contribute to potent inhibition of the cell cycle, but no induction of apoptosis by the simultaneous blockade of the pathways with TAM67 and LY294002 in H1299 NSCLC cells.

We showed some synergistic effects of LY294002 and TAM67 on the phosphorylation of Akt (Ser473) that may be associated with growth inhibition. Leaner *et al* (2005) reported that c-Jun upregulates the expression of p75-Ras-GRF1, a guanine-nucleotide exchange factor (GEF) that results in an increase in GTP-Ras and PI3K activity . Therefore, we determined whether the induction of TAM67 affected the expression of p75-Ras-GRF1 protein. We did not observe significant change in the p75-Ras-GRF1 expression (data not shown). We speculate that other c-Jun/AP-1 target proteins are involved in decreased phosphorylation of Akt by TAM67 under the treatment of LY294002.

One of the hallmark properties of transformed cells and cancer cells is that they are capable of anchorage-independent growth in culture systems, and this property correlates very well with their *in vivo* oncogenic potential (Reed, 1999; Frisch and Screaton, 2001; Grossmann, 2002; Wang, 2004). Maeno *et al* (2006) reported that deregulated c-Jun expression was involved in the acquisition of anchorage independence in human lung carcinogenesis . Activated PI3K signalling plays a critical role in protecting cells from anoikis by inactivating certain key apoptotic molecules and simultaneously enhancing anchorage-independent cell cycle progression by inhibiting the cyclin inhibitors and enhancing certain CDK activity (Wang, 2004). The inhibition of anchorage-independent growth in H1299 cells by TAM67 and LY294002 that we observed is in line with these reports.

In conclusion, the results of this study suggest that AP-1 and PI3K/Akt pathways play an essential role for the growth of some NSCLC cells. Further investigations of the involved pathways in NSCLC cells and tissues are warranted to elucidate the molecular mechanisms of NSCLC growth and may ultimately help developing an effective therapeutic strategy for treating this cancer.
